# Conditional particle filters with diffuse initial distributions

**DOI:** 10.1007/s11222-020-09975-1

**Published:** 2021-03-03

**Authors:** Santeri Karppinen, Matti Vihola

**Affiliations:** Department of Mathematics and Statistics, University of Jyväskylä, 40014 Jyväskylä, Finland

**Keywords:** Adaptive Markov chain Monte Carlo, Bayesian inference, Compartment model, Conditional particle filter, Diffuse initialisation, Hidden Markov model, Smoothing, State space model

## Abstract

Conditional particle filters (CPFs) are powerful smoothing algorithms for general nonlinear/non-Gaussian hidden Markov models. However, CPFs can be inefficient or difficult to apply with diffuse initial distributions, which are common in statistical applications. We propose a simple but generally applicable auxiliary variable method, which can be used together with the CPF in order to perform efficient inference with diffuse initial distributions. The method only requires simulatable Markov transitions that are reversible with respect to the initial distribution, which can be improper. We focus in particular on random walk type transitions which are reversible with respect to a uniform initial distribution (on some domain), and autoregressive kernels for Gaussian initial distributions. We propose to use online adaptations within the methods. In the case of random walk transition, our adaptations use the estimated covariance and acceptance rate adaptation, and we detail their theoretical validity. We tested our methods with a linear Gaussian random walk model, a stochastic volatility model, and a stochastic epidemic compartment model with time-varying transmission rate. The experimental findings demonstrate that our method works reliably with little user specification and can be substantially better mixing than a direct particle Gibbs algorithm that treats initial states as parameters.

**Supplementary Information:**

The online version contains supplementary material available at 10.1007/s11222-020-09975-1.

## Introduction

In statistical applications of general state space hidden Markov models (HMMs), commonly known also as state space models, it is often desirable to initialise the latent state of the model with a diffuse (uninformative) initial distribution (cf. Durbin and Koopman 2012). We mean by ‘diffuse’ the general scenario, where the first marginal of the smoothing distribution is highly concentrated relative to the prior of the latent Markov chain, which may also be improper.

The conditional particle filter (CPF) (Andrieu et al. 2010), and in particular its backward sampling variants (Whiteley 2010; Lindsten et al. 2014), has been found to provide efficient smoothing even with long data records, both empirically (e.g. Fearnhead and Künsch 2018) and theoretically (Lee et al. 2020). However, a direct application of the CPF to a model with a diffuse initial distribution will lead to poor performance, because most of the initial particles will ultimately be redundant, as they become drawn from highly unlikely regions of the state space.

There are a number of existing methods which can be used to mitigate this inefficiency. For simpler settings, it is often relatively straightforward to design proposal distributions that lead to an equivalent model, which no longer has a diffuse initial distribution. Indeed, if the first filtering distribution is already informative, its analytical approximation may be used directly as the first proposal distribution. The iteratively refined look-ahead approach suggested by Guarniero et al. (2017) extends to more complicated settings, but can require careful tuning for each class of problems.

We aim here for a general approach, which does not rely on any problem-specific constructions. Such a general approach which allows for diffuse initial conditions with particle Markov chain Monte Carlo (MCMC) is to include the initial latent state of the HMM as a ‘parameter’. This was suggested by Murray et al. (2013) with the particle marginal Metropolis–Hastings (PMMH). The same approach is directly applicable also with the CPF (using particle Gibbs); see Fearnhead and Meligkotsidou (2016), who discuss general approaches based on augmentation schemes.

Our approach may be seen as an instance of the general ‘pseudo-observation’ framework of Fearnhead and Meligkotsidou (2016), but we are unaware of earlier works about the specific class of methods we focus on here. Indeed, instead of building the auxiliary variable from the conjugacy perspective as Fearnhead and Meligkotsidou (2016), our approach is based on Markov transitions that are reversible with respect to the initial measure of the HMM. This approach may be simpler to understand and implement in practice, and is very generally applicable. We focus here on two concrete cases: the ‘diffuse Gaussian‘ case, where the initial distribution is Gaussian with a relatively uninformative covariance matrix, and the ‘fully diffuse‘ case, where the initial distribution is uniform. We suggest online adaptation mechanisms for the parameters, which make the methods easy to apply in practice.

We start in Sect. 2 by describing the family of models we are concerned with, and the general auxiliary variable initialisation CPF that underlies all of our developments. We present the practical methods in Sect. 3. Section 4 reports experiments of the methods with three academic models and concludes with a realistic inference task related to modelling the COVID-19 epidemic in Finland. We conclude with a discussion in Sect. 5.

## The model and auxiliary variables

Our main interest is with HMMs having a joint smoothing distribution $\pi$ of the following form:

1 $$\begin{aligned} \pi (x_{1:T}) \propto p(x_1)p(y_1\mid x_1)\prod _{k=2}^{T} p(x_k \mid x_{k-1})p(y_k \mid x_k), \end{aligned}$$

where $\ell$:*u* denotes the sequence of integers from $\ell$ to *u* (inclusive), $x_{1:T}$ denotes the latent state variables, and $y_{1:T}$ the observations. Additionally, $\pi$ may depend on (hyper)parameters $\theta$, the dependence on which we omit for now, but return to later, in Sect. 3.4.

For the convenience of notation, and to allow for some generalisations, we focus on the Feynman–Kac form of the HMM smoothing problem (cf. Del Moral 2004), where the distribution of interest $\pi$ is represented in terms of a $\sigma$-finite measure $M_1({\mathrm {d}}x_1)$ on the state space ${\mathsf {X}}$, Markov transitions $M_2,\ldots ,M_T$ on ${\mathsf {X}}$ and potential functions $G_k:{\mathsf {X}}^k\rightarrow [0,\infty )$ so that

2 $$\begin{aligned} \pi ({\mathrm {d}}x_{1:T}) \propto M_1({\mathrm {d}}x_1)G_1(x_1) \prod _{k = 2}^{T} M_k(x_{k-1}, {\mathrm {d}}x_k )G_k(x_{1:k}). \end{aligned}$$

The classical choice, the so-called ‘bootstrap filter’ (Gordon et al. 1993), corresponds to $M_1({\mathrm {d}}x_1) = p(x_1) {\mathrm {d}}x_1$ and $M_k(x_{k-1}, {\mathrm {d}}x_k) = p(x_k\mid x_{k-1}) {\mathrm {d}}x_k$, where ‘${\mathrm {d}}x$’ stands for the Lebesgue measure on ${\mathsf {X}}={\mathbb {R}}^d$, and $G_k(x_{1:k}) = p(y_k\mid x_k)$, but other choices with other ‘proposal distributions’ $M_k$ are also possible. Our main focus is when $M_1$ is diffuse with respect to the first marginal of $\pi$. We stress that our method accomodates also improper $M_1$, such as the uniform distribution on $\mathbb {R}^d$, as long as (2) defines a probability.

The key ingredient of our method is an auxiliary Markov transition, *Q*, which we can simulate from, and which satisfies the following:

### Assumption 1

($M_1$-reversibility) The Markov transition probability *Q* is reversible with respect to the $\sigma$-finite measure $M_1$, or $M_1$-reversible, if

3 $$\begin{aligned}&\int M_1({\mathrm {d}}x_0)Q(x_0, {\mathrm {d}}x_1) \mathbf {1}(x_0\in A,x_1\in B)\nonumber \\&\quad = \int M_1({\mathrm {d}}x_1)Q(x_1, {\mathrm {d}}x_0) \mathbf {1}(x_0\in A,x_1\in B), \end{aligned}$$

for all measurable $A,B\subset {\mathsf {X}}$.

We discuss practical ways to choose *Q* in Sect. 3. Assuming an $M_1$-reversible *Q*, we define an augmented target distribution, involving a new ‘pseudo-state’ $x_0$ which is connected to $x_1$ by *Q*:

$$\begin{aligned}&\tilde{\pi }({\mathrm {d}}x_{0:T}) = \pi ({\mathrm {d}}x_{1:T})Q(x_1, {\mathrm {d}}x_0) \\&\propto M_1({\mathrm {d}}x_0)Q(x_0, {\mathrm {d}}x_1) G_1(x_1) \prod _{k = 2}^{T} M_k(x_{k-1}, {\mathrm {d}}x_k) G_k(x_{1:k}). \end{aligned}$$

It is clear by construction that $\tilde{\pi }$ admits $\pi$ as its marginal, and therefore, if we can sample $x_{0:T}$ from $\tilde{\pi }$, then $x_{1:T}\sim \pi$.

Our method may be viewed as a particle Gibbs (Andrieu et al. 2010) which targets $\tilde{\pi }$, regarding $x_0$ as the ‘parameter’, and $x_{1:T}$ the ‘latent state’, which are updated using the CPF. Algorithm 1 summarises the method, which we call the ‘auxiliary initialisation’ CPF (AI-CPF). Algorithm 1 determines a $\pi$-invariant Markov transition ${\dot{x}}_{1:T} \rightarrow \tilde{X}_{1:T}^{(B_{1:T})}$; the latter output of the algorithm will be relevant later, when we discuss adaptation.
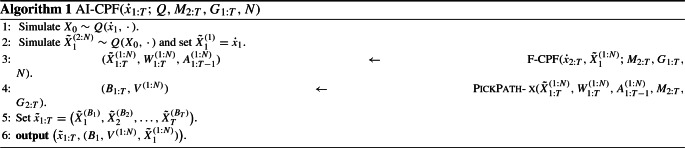


Line 1 of Algorithm 1 implements a Gibbs step sampling $X_0$ conditional on $X_{1:T}={\dot{x}}_{1:T}$, and lines 2–4 implement together a CPF targeting the conditional of $X_{1:T}$ given $X_0$. Line 3 runs what we call a ‘forward’ CPF, which is just a standard CPF conditional on the first state particles $X_{1}^{(1:N)}$, detailed in Algorithm 2. Line 4 refers to a call of $\textsc {PickPath-AT}$ (Algorithm 3) for ancestor tracing as in the original work of Andrieu et al. (2010), or $\textsc {PickPath-BS}$ (Algorithm 4) for backward sampling (Whiteley 2010). $\mathrm {Categ}(w^{(1:N)})$ stands for the categorical distribution, that is, $A \sim \mathrm {Categ}(w^{(1:N)})$ if $\Pr (A=i) = w^{(i)}$.
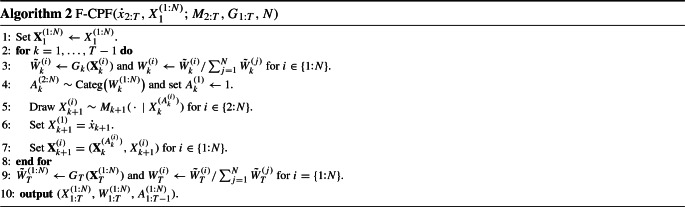


The ancestor tracing variant can be used when the transition densities are unavailable. However, our main interest here is with backward sampling, summarised in Algorithm 4 in the common case where the potentials only depend on two consecutive states, that is, $G_k(x_{1:k}) = G_k(x_{k-1:k})$, and the transitions admit densities $M_k(x_{k-1},{\mathrm {d}}x_k) = M_k(x_{k-1},x_k) {\mathrm {d}}x_k$ with respect to some dominating $\sigma$-finite measure ‘${\mathrm {d}}x_k$’.
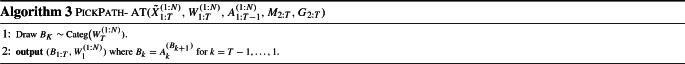

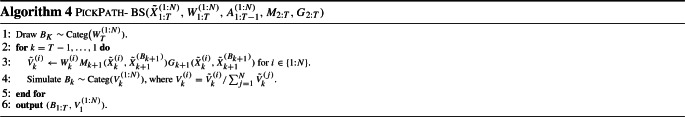


We conclude with a brief discussion on the general method of Algorithm 1.

(i) We recognise that Algorithm 1 is not new per se, in that it may be viewed just as a particle Gibbs applied for a specific auxiliary variable model. However, we are unaware of Algorithm 1 being presented with the present focus: with an $M_1$-reversible *Q*, and allowing for an improper $M_1$.
(ii) Algorithm 1 may be viewed as a generalisation of the standard CPF. Indeed, taking $Q(x_0,{\mathrm {d}}x_1) = M_1({\mathrm {d}}x_1)$ in Algorithm 1 leads to the standard CPF. Note that Line 1 is redundant in this case, but is necessary in the general case.
(iii) In the case $T=1$, Line 3 of Algorithm 1 is redundant, and the algorithm resembles certain multiple-try Metropolis methods (cf. Martino 2018) and has been suggested earlier by Mendes et al. (2015).
(iv) Algorithm 2 is formulated using multinomial resampling, for simplicity. We note that any other unbiased resampling may be used, as long as the conditional resampling is designed appropriately; see Chopin and Singh (2015).

The ‘CPF generalisation’ perspective of Algorithm 1 may lead to other useful developments; for instance, one could imagine the approach to be useful with the CPF applied for static (non-HMM) targets, as in sequential Monte Carlo samplers (Del Moral et al. 2006). The aim of the present paper is, however, to use Algorithm 1 with diffuse initial distributions.

## Methods for diffuse initialisation of conditional particle filters

To illustrate the typical problem that arises with a diffuse initial distribution $M_1$, we examine a simple noisy AR(1) model:

4 $$\begin{aligned}&x_{k+1}= \rho x_{k} + \eta _k, \eta _k \sim N(0, \sigma _x^2)\nonumber \\&y_{k} = x_k + \epsilon _k, \epsilon _k \sim N(0, \sigma _y^2), \end{aligned}$$

for $$k\ge 1$$, $$x_1 \sim N(0, \sigma _1^2)$$, $$M_1({\mathrm {d}}x_1) = p(x_1) {\mathrm {d}}x_1$$, $$M_k(x_{k-1}, {\mathrm {d}}x_k) = p(x_k\mid x_{k-1}) {\mathrm {d}}x_k$$ and $$G_k(x_{1:k}) = p(y_k\mid x_k)$$.

**Fig. 1:**
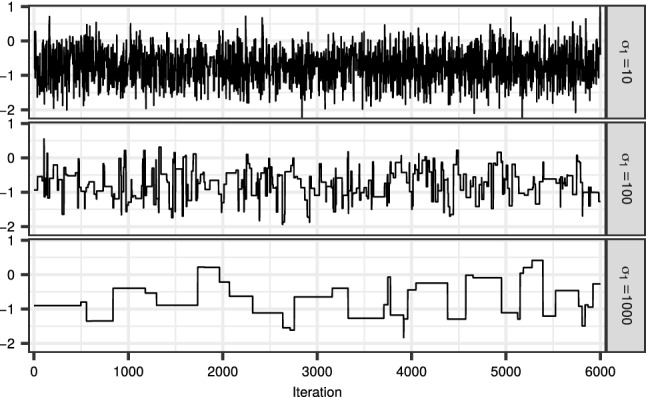
Traceplot of the initial state of the noisy AR(1) model, using the CPF with 16 particles and backward sampling with $\sigma _{1} = 10$ (top), 100 (middle) and 1000 (bottom)

We simulated a dataset of length $T=50$ from this model with $x_1 = 0$, $\rho = 0.8$ and $\sigma _x = \sigma _y = 0.5$. We then ran 6000 iterations of the CPF with backward sampling (CPF-BS) with $\sigma _1 \in \{10, 100, 1000\}$; that is, Algorithm 1 with $Q(x_0,\,\cdot \,) = M_1(\,\cdot \,)$ together with Algorithm 4, and discarded the first 1000 iterations as burn-in. For each value of $\sigma _1$, we monitored the efficiency of sampling $x_1$. Figure 1 displays the resulting traceplots. The estimated integrated autocorrelation times (${\mathrm {IACT}}$) were approximately 3.75, 28.92 and 136.64, leading to effective sample sizes (${\mathrm {n}}_{\mathrm {eff}}$) of 1600, 207 and 44, respectively. This demonstrates how the performance of the CPF-BS deteriorates as the initial distribution of the latent state becomes more diffuse.

### Diffuse Gaussian initialisation

In the case that $M_1$ in (2) is Gaussian with mean $\mu$ and covariance $\varSigma$, we can construct a Markov transition function that satisfies (3) using an autoregressive proposal similar to ‘preconditioning’ in the Crank-Nicolson algorithm (cf. Cotter et al. 2013). This proposal comes with a parameter $\beta \in (0, 1]$, so we denote this kernel by $Q_{\beta }^{\mathrm {AR}}$. A variate $Z \sim Q_{\beta }^{\mathrm {AR}}(x, \,\cdot \,)$ can be drawn simply by setting

5 $$\begin{aligned} Z = \sqrt{1 - \beta ^2}(x - \mu ) + \beta W + \mu , \end{aligned}$$

where $W \sim N(0, \varSigma )$. We refer to Algorithm 1 with $Q = Q_{\beta }^{\mathrm {AR}}$ as the diffuse Gaussian initialisation CPF (DGI-CPF). In the special case $\beta = 1$, we have $Q_{1}^{\mathrm {AR}} = M_1$, and so the DGI-CPF is equivalent with the standard CPF.

### Fully diffuse initialisation

Suppose that $M_1({\mathrm {d}}x) = M_1(x) {\mathrm {d}}x$ where $M_1(x)\equiv 1$ is a uniform density on ${\mathsf {X}}={\mathbb {R}}^d$. Then, any symmetric transition *Q* satisfies $M_1$-reversibility. In this case, we suggest to use $Q_{C}^{\mathrm {RW}}(x,{\mathrm {d}}y) = q_{C}^{\mathrm {RW}}(x,y){\mathrm {d}}y$ with a multivariate normal density $q_{C}^{\mathrm {RW}}(x,y) = N(y; x, C)$, with covariance $C\in {\mathbb {R}}^{d\times d}$. In case of constraints, that is, a non-trivial domain $D\subset {\mathbb {R}}^d$, we have $M_1 = 1(x\in D)$. Then, we suggest to use a Metropolis–Hastings type transition probability:

$$\begin{aligned} Q_{C}^{\mathrm {RW}}(x,{\mathrm {d}}y)= & {} q_{C}^{\mathrm {RW}}(x,y) \min \bigg \{ 1, \frac{M_1(y)}{M_1(x)} \bigg \} {\mathrm {d}}y \\&+ \,\delta _x({\mathrm {d}}y) r(x), \end{aligned}$$

where $r(x)\in [0,1]$ is the rejection probability. This method works, of course, with arbitrary $M_1$, but our focus is with a diffuse case, where the domain *D* is regular and large enough, so that rejections are rare. We stress that also in this case, $M_1(x) = 1(x\in D)$ may be improper. We refer to Algorithm 1 with $Q_{C}^{\mathrm {RW}}$ as the ‘fully diffuse initialisation’ CPF (FDI-CPF).

We note that whenever $M_1$ can be evaluated pointwise, the FDI-CPF can always be applied, by considering the modified Feynman–Kac model $\tilde{M}_1\equiv 1$ and $\tilde{G}_1(x) = M_1(x) G_1(x)$. However, when $M_1$ is Gaussian, the DGI-CPF can often lead to a more efficient method. As with standard random walk Metropolis algorithms, choosing the covariance $C\in {\mathbb {R}}^{d\times d}$ is important for the efficiency of the FDI-CPF.

### Adaptive proposals

Finding a good autoregressive parameter of $Q_{\beta }^{\mathrm {AR}}$ or the covariance parameter of $Q_{C}^{\mathrm {RW}}$ may be time-consuming in practice. Inspired by the recent advances in adaptive MCMC (cf. Andrieu and Thoms 2008; Vihola 2020), it is natural to apply adaptation also with the (iterated) AI-CPF. Algorithm 5 summarises a generic adaptive AI-CPF (AAI-CPF) using a parameterised family $\{Q_\zeta \}_{\zeta \in {\mathsf {Z}}}$ of $M_1$-reversible proposals, with parameter $\zeta$.
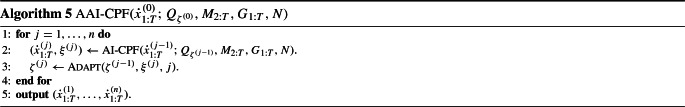


The function $\textsc {Adapt}$ implements the adaptation, which typically leads to $\zeta ^{(j)} \rightarrow \zeta ^*$, corresponding to a well-mixing configuration. We refer to the instances of the AAI-CPF with the AI-CPF step corresponding to the DGI-CPF and the FDI-CPF as the adaptive DGI-CPF and FDI-CPF, respectively.

We next focus on concrete adaptations which may be used within our framework. In the case of the FDI-CPF, Algorithm 6 implements a stochastic approximation variant (Andrieu and Moulines 2006) of the adaptive Metropolis covariance adaptation of Haario et al. (2001).
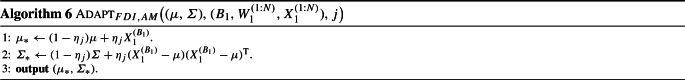


Here, $\eta _j$ are step sizes that decay to zero, $\zeta _j = (\mu _j,\varSigma _j)$ the estimated mean and covariance of the smoothing distribution, respectively, and $Q_\zeta = Q_{c \varSigma }^{\mathrm {RW}}$ where $c>0$ is a scaling factor of the covariance $\varSigma$. In the case of random walk Metropolis, this scaling factor is usually taken as $2.38^2/d$ (Gelman et al. 1996), where *d* is the state dimension of the model. In the present context, however, the optimal value of $c > 0$ appears to depend on the model and on the number of particles *N*. This adaptation mechanism can be used both with PickPath-AT and with PickPath-BS, but may require some manual tuning to find a suitable $c>0$.

Algorithm 7 details another adaptation for the FDI-CPF, which is intended to be used together with PickPath-BS only. Here, $\zeta _j = (\mu _j,\varSigma _j, \delta _j)$ contains the estimated mean, covariance and the scaling factor, and $Q_\zeta = Q_{C(\zeta )}^{\mathrm {RW}}$, where $C(\zeta ) = e^\delta \varSigma$.
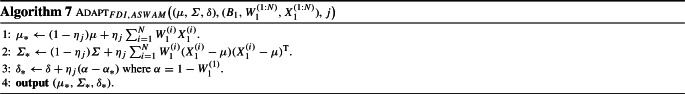

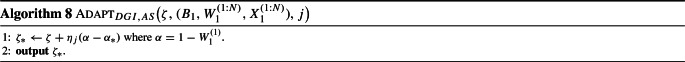


This algorithm is inspired by a Rao–Blackwellised variant of the adaptive Metropolis within adaptive scaling method (cf. Andrieu and Thoms 2008), which is applied with standard random walk Metropolis. We use all particles with their backward sampling weights to update the mean $\mu$ and covariance $\varSigma$, and an ‘acceptance rate’ $\alpha$, that is, the probability that the first coordinate of the reference trajectory is not chosen. Recall that after the AI–CPF in Algorithm 5 has been run, the first coordinate of the reference trajectory and its associated weight reside in the first index of the particle and weight vectors contained in $\xi ^{(j)}$.

The optimal value of the acceptance rate parameter ${\alpha _{*}}$ is typically close to one, in contrast with random walk Metropolis, where ${\alpha _{*}}\in [0.234,0.44]$ are common (Gelman et al. 1996). Even though the optimal value appears to be problem-dependent, we have found empirically that $0.7\le {\alpha _{*}}\le 0.9$ often leads to reasonable mixing. We will show empirical evidence for this finding in Sect. 4.

Algorithm 8 describes a similar adaptive scaling type mechanism for tuning $\beta = {\mathrm {logit}}^{-1}(\zeta )$ in the DGI-CPF, with $Q_\zeta = Q_{\beta }^{\mathrm {AR}}$. The algorithm is most practical with PickPath-BS.

We conclude this section with a consistency result for Algorithm 5, using the adaptation mechanisms in Algorithms 6 and 7. In Theorem 1, we denote $(\mu _j,\varSigma _j) = \zeta _j$ in the case of Algorithm 6, and $(\mu _j,\varSigma _j,\delta _j) = \zeta _j$ with Algorithm 7.

#### Theorem 1

Suppose *D* is a compact set, a uniform mixing condition (Assumption 2 in Appendix A) holds, and there exists an $\epsilon >0$ such that for all $j\ge 1$, the smallest eigenvalue $\lambda _{\min }(\varSigma _j)\ge \epsilon$, and with Algorithm 7 also $\delta _j\in [\epsilon ,\epsilon ^{-1}]$. Then, for any bounded function $f:{\mathsf {X}}\rightarrow \infty$,

$$\begin{aligned} \frac{1}{n}\sum _{k=1}^n f({\dot{x}}_{1:T}^{(k)}) \xrightarrow {n\rightarrow \infty } \pi (f).\qquad \text {almost surely.} \end{aligned}$$

The proof of Theorem 1 is given in Appendix A. The proof is slightly more general, and accomodates for instance *t*-distributed instead of Gaussian proposals for the FDI-CPF. We note that the latter stability condition, that is, existence of the constant $\epsilon >0$, may be enforced by introducing a ‘rejection’ mechanism in the adaptation; see the end of Appendix A. However, we have found empirically that the adaptation is stable also without such a stabilisation mechanism.

### Use within particle Gibbs

Typical application of HMMs in statistics involves not only smoothing, but also inference of a number of ‘hyperparameters’ $\theta$, with prior density $\mathrm {pr}(\theta )$, and with

6 $$\begin{aligned} \gamma _\theta (x_{1:T})&= p(y_{1:T},x_{1:T}\mid \theta ) \\&= M_1(x_1) G_1^{(\theta )}(x_1) \prod _{k=2}^T M_k^{(\theta )}(x_{k-1},x_k) G_k^{(\theta )}(x_{k-1},x_k). \nonumber \end{aligned}$$

The full posterior, $\check{\pi }(\theta , x_{1:T}) \propto \mathrm {pr}(\theta ) \gamma _\theta (x_{1:T})$ may be inferred with the particle Gibbs (PG) algorithm of Andrieu et al. (2010). (We assume here that $M_1$ is diffuse, and thereby independent of $\theta$.)

The PG alternates between (Metropolis-within-)Gibbs updates for $\theta$ conditional on $x_{1:T}$, and CPF updates for $x_{1:T}$ conditional on $\theta$. The (A)AI-CPF applied with $M_{2:T}^{(\theta )}$ and $G_{1:T}^{(\theta )}$ may be used as a replacement of the CPF steps in a PG. Another adaptation, independent of the AAI-CPF, may be used for the hyperparameter updates (cf. Vihola 2020).

Algorithm 9 summarises a generic adaptive PG with the AAI-CPF. Line 2 involves an update of $\theta ^{(j-1)}$ to $\theta ^{(j)}$ using transition probabilities $K_{\zeta _\theta }(\,\cdot \,, \,\cdot \,\mid x_{1:T})$ which leave $\check{\pi }(\theta \mid x_{1:T})$ invariant, and Line 3 is (optional) adaptation. This could, for instance, correspond to the robust adaptive Metropolis algorithm (RAM) (Vihola 2012). Lines 4 and 5 implement the AAI-CPF. Note that without Lines 3 and 5, Algorithm 9 determines a $\check{\pi }$-invariant transition rule.
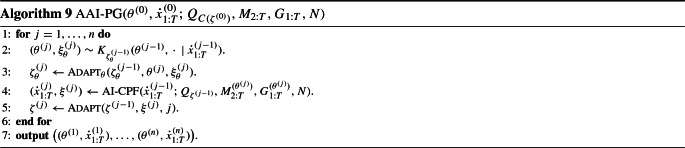


## Experiments

In this section, we study the application of the methods presented in Sect. 3 in practice. Our focus will be on the case of the bootstrap filter, that is, $M_1({\mathrm {d}}x_1) = p(x_1) {\mathrm {d}}x_1$, $M_k(x_{k-1}, {\mathrm {d}}x_k) = p(x_k\mid x_{k-1}) {\mathrm {d}}x_k$ and $G_k(x_{1:k}) = p(y_k\mid x_k)$.

We start by investigating two simple HMMs: the noisy random walk model (RW), that is, (4) with $\rho = 1$, and the following stochastic volatility (SV) model:

7 $$\begin{aligned}&x_{k+1} = x_{k} + \eta _k, \nonumber \\&y_{k} = e^{x_k}\epsilon _k, \end{aligned}$$

with $x_1 \sim N(0, \sigma _1^2)$, $\eta _k \sim N(0, \sigma _x^2)$ and $\epsilon _k \sim N(0, \sigma _y^2)$. In Sect. 4.3, we study the dependence of the method with varying dimension, with a static multivariate normal model. We conclude in Sect. 4.4 by applying our methods in a realistic inference problem related to modelling the COVID-19 epidemic in Finland.

### Comparing DGI-CPF and CPF-BS

We first studied how the DGI-CPF performs in comparison to the CPF-BS when the initial distributions of the RW and SV model are diffuse. Since the efficiency of sampling is affected by both the values of the model parameters (cf. Fig. 1) and the number of particles *N*, we experimented with a range of values $N \in \{8, 16, 32, 64, 128, 256, 512\}$ for which we applied both methods with $n = 10000$ iterations plus 500 burn-in. We simulated data from both the RW and SV models with $T = 50$, $x_{1} = 0$, $\sigma _y = 1$ and varying $\sigma _x \in \{0.01, 0.05, 0.1, 0.5, 1, 2, 5, 10, 20, 50, 100, 200\}$. We then applied both methods for each dataset with the corresponding $\sigma _x$, but with varying $\sigma _1 \in \{10, 50, 100, 200, 500, 1000\}$, to study the sampling efficiency under different parameter configurations ($\sigma _x$ and $\sigma _1$). For the DGI-CPF, we varied the parameter $\beta \in \{0.01, 0.02, \ldots , 0.99\}$. We computed the estimated integrated autocorrelation time (${\mathrm {IACT}}$) of the simulated values of $x_1$ and scaled this by the number of particles *N*. The resulting quantity, the inverse relative efficiency (${\mathrm {IRE}}$), measures the asymptotic efficiencies of estimators with varying computational costs (Glynn and Whitt 1992).

**Fig. 2:**
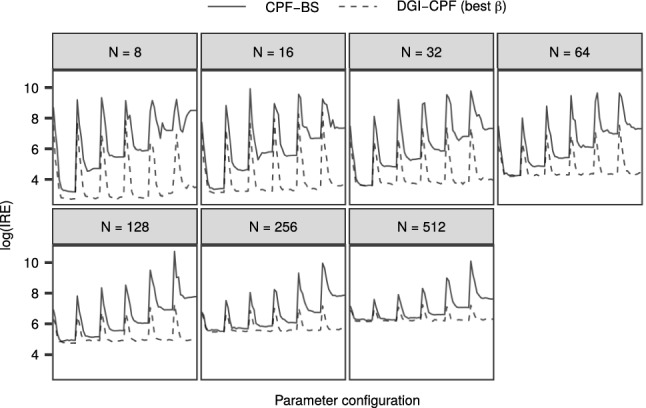
The $\log {({\mathrm {IRE}})}$ resulting from the application of the CPF-BS and the best case DGI-CPF to the RW model. The horizontal axis depicts different configurations of $\sigma _1$ and $\sigma _x$, and in each panel *N* varies

Figure 2 shows the comparison of the CPF-BS with the best DGI-CPF, that is, the DGI-CPF with the $\beta$ that resulted in the lowest ${\mathrm {IACT}}$ for each parameter configuration and *N*.

The results indicate that with *N* fixed, a successful tuning of $\beta$ can result in greatly improved mixing in comparison with the CPF-BS. While the performance of the CPF-BS approaches that of the best DGI-CPF with increasing *N*, the difference in performance remains substantial with parameter configurations that are challenging for the CPF-BS.

The optimal *N* which minimizes the ${\mathrm {IRE}}$ depends on the parameter configuration. For ‘easy’ configurations (where ${\mathrm {IRE}}$ is small), even $N=8$ can be enough, but more ‘difficult’ configurations (where ${\mathrm {IRE}}$ is large), higher values of *N* can be optimal. Similar results for the SV model are shown in Online Resource 1 (Fig. 1), and lead to similar conclusions.

**Fig. 3:**
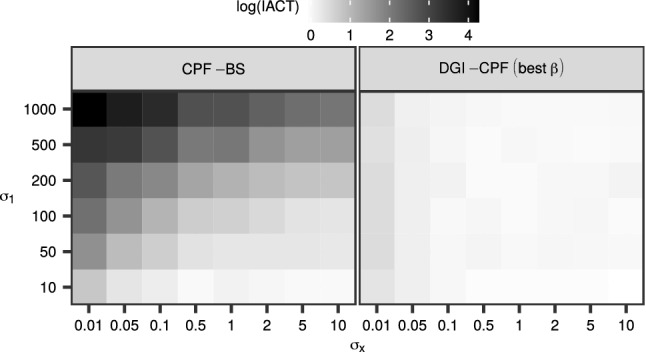
The $\log {({\mathrm {IACT}})}$ of the CPF-BS (left) and the best case DGI-CPF (right) with respect to $\sigma _1$ and $\sigma _x$ in the case of the SV model and $N = 256$

The varying ‘difficulty’ of the parameter configurations is further illustrated in Fig. 3, which shows the $\log {({\mathrm {IACT}})}$ for the SV model with $N = 256$ particles. The CPF-BS performed the worst when the initial distribution was very diffuse with respect to the state noise $\sigma _x$, as expected. In contrast, the well-tuned DGI-CPF appears rather robust with respect to changing parameter configuration. The observations were similar with other *N*, and for the RW model; see Online Resource 1 (Fig. 2).

**Fig. 4:**
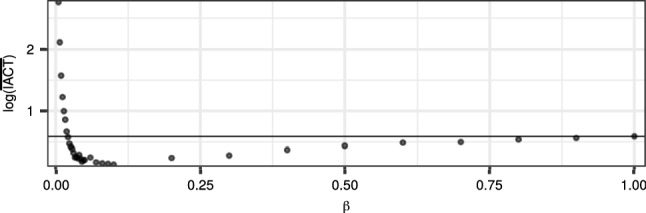
The logarithm of the mean ${\mathrm {IACT}}$ over 5 replicate runs of the DGI-CPF with respect to varying $\beta$. The dataset was simulated from the SV model with parameters $\sigma _x = 1$ and $\sigma _{1} = 50$ and fixed in each replicate run of the algorithm. *N* was set to 128. The horizontal line depicts the performance of the CPF-BS

The results in Figs. 2 and 3 illustrate the potential of the DGI-CPF, but are overly optimistic because in practice, the $\beta$ parameter of the DGI-CPF cannot be chosen optimally. Indeed, the choice of $\beta$ can have a substantial effect on the mixing. Figure 4 illustrates this in the case of the SV model by showing the logarithm of the mean ${\mathrm {IACT}}$ over replicate runs of the DGI-CPF, for a range of $\beta$. Here, a $\beta$ of approximately 0.125 seems to yield close to optimal performance, but if the $\beta$ is chosen too low, the sampling efficiency is greatly reduced, rendering the CPF-BS more effective.

**Fig. 5:**
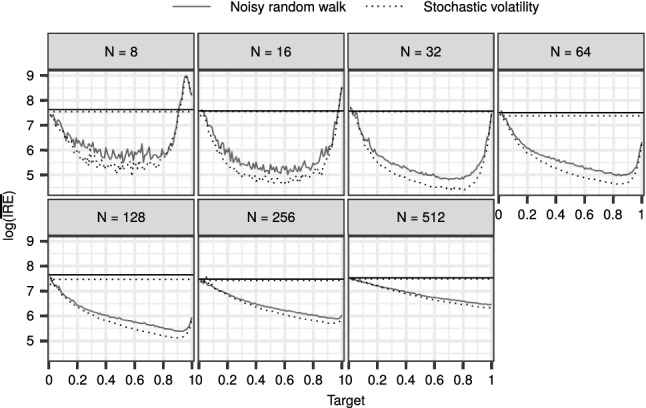
The logarithm of the mean ${\mathrm {IRE}}$ over the parameter configurations with the adaptive DGI-CPF and varying target acceptance rates. The horizontal lines depict the mean performance of the CPF-BS

This highlights the importance of choosing an appropriate value for $\beta$, and motivates our adaptive DGI-CPF, that is, Algorithm 5 together with Algorithm 8. We explored the effect of the target acceptance rate ${\alpha _{*}}\in \{0.01, 0.02, \ldots , 1\}$, with the same datasets and parameter configurations as before. Figure 5 summarises the results for both the SV and RW models, in comparison with the CPF-BS. The results indicate that with a wide range of target acceptance rates, the adaptive DGI-CPF exhibits improved mixing over the CPF-BS. When *N* increases, the optimal values for ${\alpha _{*}}$ appear to tend to one. However, in practice, we are interested in a moderate *N*, for which the results suggest that the best candidates for values of ${\alpha _{*}}$ might often be found in the range from 0.7 to 0.9.

For the CPF-BS, the mean ${\mathrm {IRE}}$ is approximately constant, which might suggest that the optimal number of particles is more than 512. In contrast, for an appropriately tuned DGI-CPF, the mean ${\mathrm {IRE}}$ is optimised by $N = 32$ in this experiment.

### Comparing FDI-CPF and particle Gibbs

Next, we turn to study a fully diffuse initialisation. In this case, $M_1$ is improper, and we cannot use the CPF directly. Instead, we compare the performance of the adaptive FDI-CPF with what we call the diffuse particle Gibbs (DPG-BS) algorithm. The DPG-BS is a standard particle Gibbs algorithm, where the first latent state $x_1$ is regarded as a ‘parameter’, that is, the algorithm alternates between the update of $x_1$ conditional on $x_{2:T}$ using a random walk Metropolis-within-Gibbs step, and the update of the latent state variables $x_{2:T}$ conditional on $x_1$ using the CPF-BS. We also adapt the Metropolis-within-Gibbs proposal distribution $Q_{\mathrm {DPG}}$ of the DPG-BS, using the RAM algorithm (cf. Vihola 2020). For further details regarding our implementation of the DPG-BS, see Appendix B.

We used a similar simulation experiment as with the adaptive DGI-CPF in Sect. 4.1, but excluding $\sigma _1$, since the initial distribution was now fully diffuse. The target acceptance rates in the FDI-CPF with the ASWAM adaptation were again varied in ${\alpha _{*}}\in \{0.01, 0.02, \ldots , 1\}$ and the scaling factor in the AM adaptation was set to $c = 2.38^2$. In the DPG-BS, the target acceptance rate for updates of the initial state using the RAM algorithm was fixed to 0.441 following Gelman et al. (1996).

**Fig. 6:**
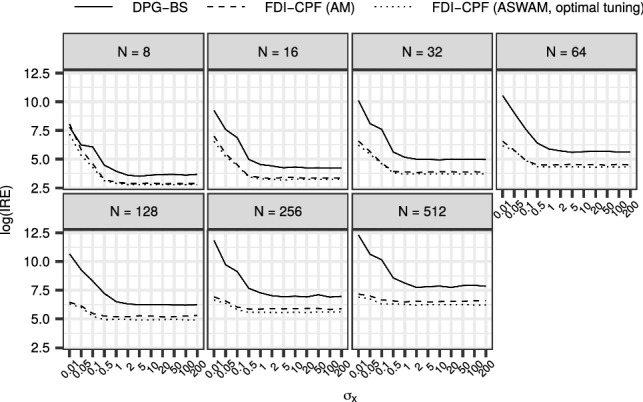
The $\log {({\mathrm {IRE}})}$ for the DPG-BS, FDI-CPF with the AM adaptation and the best case FDI-CPF with the ASWAM adaptation to the datasets generated with varying $\sigma _x$ from the RW model

Figure 6 shows results with the RW model for the DPG-BS, the FDI-CPF with the AM adaptation, and the FDI-CPF with the ASWAM adaptation using the best value for ${\alpha _{*}}$. The FDI-CPF variants appear to perform better and improve upon the performance of the DPG-BS especially with small $\sigma _x$. Similar to Figs. 2 and 3, the optimal *N* minimizing the ${\mathrm {IRE}}$ depends on the value of $\sigma _x$: smaller values of $\sigma _x$ call for higher number of particles.

The performance of the adaptive FDI-CPF appears similar regardless of the adaptation used, because the chosen scaling factor $c = 2.38^2$ for a univariate model was close to the optimal value found by the ASWAM variant in this example. We experimented also with $c = 1$, which led to less efficient AM, in the middle ground between the ASWAM and the DPG-BS.

The ${\mathrm {IACT}}$ for the DPG-BS stays approximately constant with increasing *N*, which results in a $\log {({\mathrm {IRE}})}$ that increases roughly by a constant as *N* increases. This is understandable, because in the limit as $N\rightarrow \infty$, the CPF-BS (within the DPG-BS) will correspond to a Gibbs step, that is, a perfect sample of $x_{2:T}$ conditional on $x_1$. Because of the strong correlation between $x_1$ and $x_2$, even an ‘ideal’ Gibbs sampler remains inefficient, and the small variation seen in the panels for the DPG-BS is due to sampling variability. The results for the SV model, with similar findings, are shown in Online Resource 1 (Fig. 3).

**Fig. 7:**
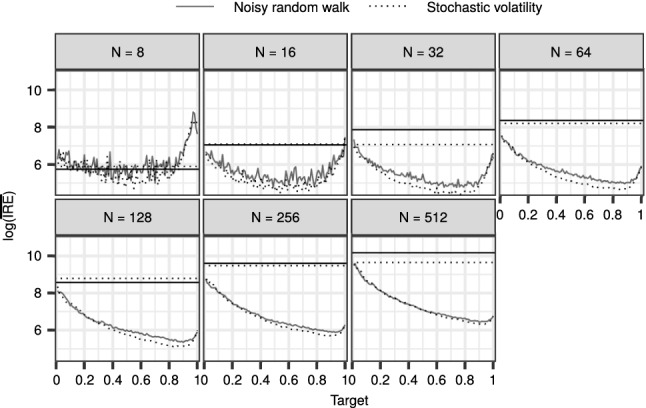
A comparison of the FDI-CPF with the ASWAM adaptation against the DPG-BS. The horizontal axis shows the target acceptance rate ${\alpha _{*}}$ used in the adaptive FDI-CPF. The logarithm of the mean ${\mathrm {IRE}}$ on the vertical axis is computed over the different $\sigma _x$ values. The black horizontal lines show the performance with the DPG-BS

Figure 7 shows the logarithm of the mean ${\mathrm {IRE}}$ of the FDI-CPF with the ASWAM adaptation with respect to varying target acceptance rate ${\alpha _{*}}$. The results are reminiscent of Fig. 5 and show that with a moderate fixed *N*, the FDI-CPF with the ASWAM adaptation outperforms the DPG-BS with a wide range of values for ${\alpha _{*}}$. The optimal value of ${\alpha _{*}}$ seems to tend to one as *N* increases, but again, we are mostly concerned with moderate *N*. For a well-tuned FDI-CPF the minimum mean ${\mathrm {IRE}}$ is found when *N* is roughly between 32 and 64.

### The relationship between state dimension, number of particles and optimal target acceptance rate

A well chosen value for the target acceptance rate ${\alpha _{*}}$ appears to be key for obtaining good performance with the adaptive DGI-CPF and the FDI-CPF with the ASWAM adaptation. In Sects. 4.1–4.2, we observed a relationship between *N* and the optimal target acceptance rate, denoted here by $\alpha _{\mathrm {opt}}$, with two univariate HMMs. It is expected that $\alpha _{\mathrm {opt}}$ is generally somewhat model-dependent, but in particular, we suspected that the methods might behave differently with models of different state dimension *d*.

In order to study the relationship between *N*, *d* and $\alpha _{\mathrm {opt}}$ in more detail, we considered a simple multivariate normal model with $T = 1$, $M_1(x) \propto 1$, and $G_1(x_1) = N(x_1; 0, \sigma I_d)$, the density of *d* independent normals. We conducted a simulation experiment with 6000 iterations plus 500 burn-in. We applied the FDI-CPF with the ASWAM adaptation with all combinations of $N \in \{2^4, 2^5, \ldots , 2^{11}\}$, ${\alpha _{*}}\in \{0.01, 0.02, \ldots , 1\}$, $\sigma \in \{1, 5, 10, 50, 100\}$, and with dimension $d \in \{1, 2, \ldots , 10\}$. Unlike before, we monitor the ${\mathrm {IACT}}$ over the samples of $x_1$ as an efficiency measure.

**Fig. 8:**
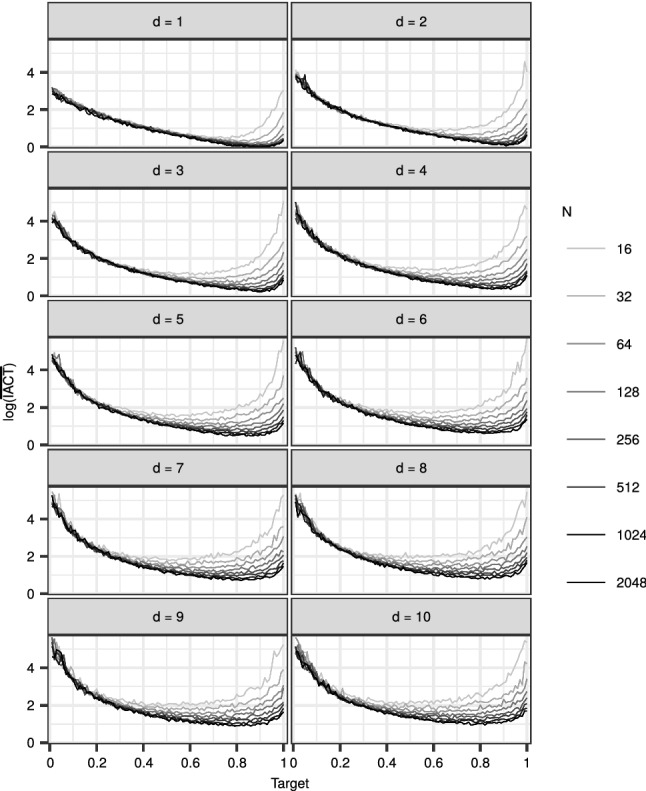
The effect of state dimension *d*, number of particles *N* and target acceptance rate ${\alpha _{*}}$ on the logarithm of the mean ${\mathrm {IACT}}$ in the multivariate normal model. The means are computed over the different $\sigma$ in the simulation experiment

Figure 8 summarises the results of this experiment. With a fixed state dimension, $\alpha _{\mathrm {opt}}$ tended towards 1 with increasing numbers of particles *N*, as observed with the RW and SV models above. With a fixed number of particles *N*, $\alpha _{\mathrm {opt}}$ appears to get smaller with increasing state dimension *d*, but the change rate appears slower with higher *d*. Again, with moderate values for *N* and *d*, the values in the range 0.7–0.9 seem to yield good performance.

**Fig. 9:**
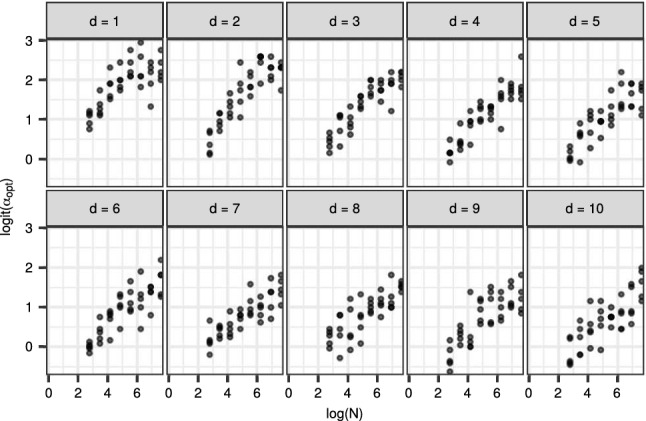
The best target acceptance rate $\alpha _{\mathrm {opt}}$ with respect to the number of particles *N* and state dimension *d* on the multivariate normal model

Figure 9 shows a different view of the same data: ${\mathrm {logit}}{(\alpha _{\mathrm {opt}})}$ is plotted with respect to $\log {(N)}$ and *d*. Here, we computed $\alpha _{\mathrm {opt}}$ by taking the target acceptance rate that produced the lowest ${\mathrm {IACT}}$ in the simulation experiment, for each value of $\sigma$, *N* and *d*. At least with moderate $\alpha _{\mathrm {opt}}$ and *N*, there appears to be a roughly linear relationship between ${\mathrm {logit}}(\alpha _{\mathrm {opt}})$ and $\log (N)$, when *d* is fixed. However, because of the lack of theoretical backing, we do not suggest to use such a simple model for choosing $\alpha _{\mathrm {opt}}$ in practice.

### Modelling the COVID-19 epidemic in Finland

Our final experiment is a realistic inference problem arising from the modelling of the progress of the COVID-19 epidemic in Uusimaa, the capital region of Finland. Our main interest is in estimating the time-varying transmission rate, or the basic reproduction number ${{\mathscr {R}}_{0}}$, which is expected to change over time, because of a number of mitigation actions and social distancing. The model consists of a discrete-time ‘SEIR’ stochastic compartment model, and a dynamic model for ${{\mathscr {R}}_{0}}$; such epidemic models have been used earlier in different contexts (e.g. Shubin et al. 2016).

We use a simple SEIR without age/regional stratification. That is, we divide the whole population ${N}_{\mathrm {pop}}$ to four separate states: susceptible (*S*), exposed (*E*), infected (*I*) and removed (*R*), so that ${N}_{\mathrm {pop}}= S + E + I + R$, and assume that ${N}_{\mathrm {pop}}$ is constant. We model the transformed ${{\mathscr {R}}_{0}}$, denoted by $\rho$, such that ${{\mathscr {R}}_{0}}= {{\mathscr {R}}_{0}}^{\mathrm {max}}{\mathrm {logit}}^{-1}(\rho )$, where ${{\mathscr {R}}_{0}}^{\mathrm {max}}$ is the maximal value for ${{\mathscr {R}}_{0}}$. The state vector of the model at time *k* is, therefore, $X_k = (S_k, E_k, I_k, R_k, \rho _k)$. One step of the SEIR is:

$$\begin{aligned} S_{k + 1}= & {} S_k - \varDelta E_{k + 1}, \\ E_{k + 1}= & {} E_k + \varDelta E_{k + 1} - \varDelta I_{k + 1}, \\ I_{k + 1}= & {} I_k + \varDelta I_{k + 1} - \varDelta R_{k + 1}, \\ R_{k + 1}= & {} R_k + \varDelta R_{k + 1}, \\ \rho _{k+1}= & {} \rho _k + \varDelta \rho _{k + 1}, \end{aligned}$$

where the increments are as distributed as follows:

$$\begin{aligned} \varDelta E_{k+1}&\sim {\mathrm {Binomial}}(S_k, p_\beta ), \ p_\beta = 1 - \exp {(- \beta _k (I_k / {N}_{\mathrm {pop}}))}, \\ \varDelta I_{k+1}&\sim {\mathrm {Binomial}}(E_k, p_a), \ p_a = 1 - \exp {(- a)}, \\ \varDelta R_{k + 1}&\sim {\mathrm {Binomial}}(I_k, p_\gamma ), \ p_\gamma = 1 - \exp {(- \gamma )}, \\ \varDelta \rho _{k + 1}&\sim {\mathrm {Normal}}(0, \sigma ^2). \end{aligned}$$

Here, $\beta _k = {{\mathscr {R}}_{0}}^{\mathrm {max}}{\mathrm {logit}}^{-1}(\rho _k) p_\gamma$ is the time-varying infection rate, and $a^{-1}$ and $\gamma ^{-1}$ are the mean incubation period and recovery time, respectively. Finally, the random walk parameter $\sigma$ controls how fast $(\rho _k)_{k \ge 2}$ can change.

The data we use in the modelling consist of the daily number of individuals tested positive for COVID-19 in Uusimaa (Finnish Institute for Health and Welfare 2020). We model the counts with a negative binomial distribution dependent on the number of infected individuals:

8 $$\begin{aligned} Y_k \sim {\mathrm {NegativeBinomial}}\left( e p_{\gamma }\dfrac{p}{1 - p}I_k, p\right) . \end{aligned}$$

Here, the parameter *e* denotes sampling effort, that is, the average proportion of infected individuals that are observed, and *p* is the failure probability of the negative binomial distribution, which controls the variability of the distribution.

In the beginning of the epidemic, there is little information available regarding the initial states, rendering the diffuse initialisation a convenient strategy. We set

9 $$\begin{aligned}&M_1(S_1, E_1, I_1, R_1, \rho _1) \nonumber \\&\quad \! =\! 1(S_1 \!+ \!E_1 \!+\! I_1 \!=\! {N}_{\mathrm {pop}}) 1(S_1, E_1, I_1 \!\ge \! 0)1(R_1 \!=\! 0), \end{aligned}$$

where the number of removed $R_1 = 0$ is justified because we assume all were susceptible to COVID-19, and that the epidemic has started very recently.

In addition to the state estimation, we are interested in estimating the parameters $\sigma$ and *p*. We assign the prior $N(-2.0, (0.3)^2)$ to $\log {(\sigma )}$ to promote gradual changes in ${{\mathscr {R}}_{0}}$, and an uninformative prior, $N(0, 10^2)$, for ${\mathrm {logit}}(p)$. The remaining parameters are fixed to ${N}_{\mathrm {pop}}= 1638469$, ${{\mathscr {R}}_{0}}^{\mathrm {max}} = 10$, $a = 1/3$, $\gamma = 1/7$ and $e = 0.15$, which are in part inspired by the values reported by the Finnish Institute for Health and Welfare.

We used the AAI-PG (Algorithm 9) with the FDI-CPF with the ASWAM adaptation, and a RAM adaptation (Vihola 2012) for $\sigma$ and *p*, (i.e. in the Lines 2–3 of Algorithm 9). The form of (9) leads to the version of the FDI-CPF discussed in Sect. 3.2 where the initial distribution is uniform with constraints. We use a random walk proposal to generate proposals $(\rho _1,E_1,I_1)\rightarrow (\rho _1^*,E_1^*,I_1^*)$, round $E_1^*$ and $I_1^*$ to the nearest integer, and then set $R_1^* = 0$ and $S_1^* = {N}_{\mathrm {pop}}- E_1^* - I_1^* - R_1^{*}$. We refer to this variant of the AAI-PG as the FDI-PG algorithm. Motivated by our findings in Sects. 4.1–4.3, we set the target acceptance rate ${\alpha _{*}}$ in the FDI-CPF (within the FDI-PG) to 0.8.

As an alternative to the FDI-PG we also used a particle Gibbs algorithm that treats $\sigma$, *p* as well as the initial states $E_1$, $I_1$ and $\rho _1$ as parameters, using the RAM to adapt the random walk proposal (Vihola 2012). This algorithm is the DPG-BS detailed in Appendix B with the difference that the parameters $\sigma$ and *p* are updated together with the initial state, and $p^{\mathrm {DPG}}$ additionally contains all terms of (6) which depend on $\sigma$ and *p*.

**Fig. 10:**
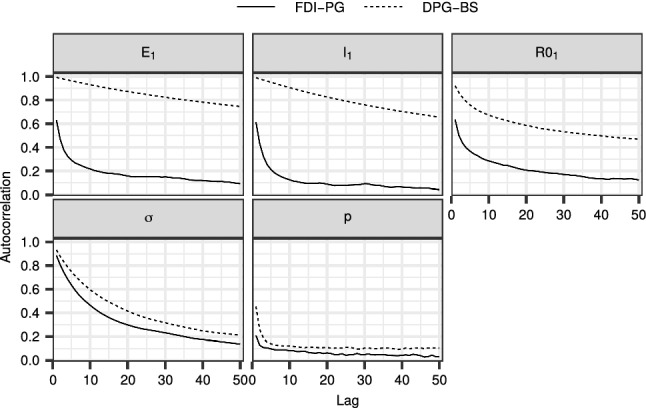
The first 50 autocorrelations for the model parameters and initial states with the FDI-PG and the DPG-BS computed after thinning the total 500000 samples to every 10th sample

**Fig. 11:**
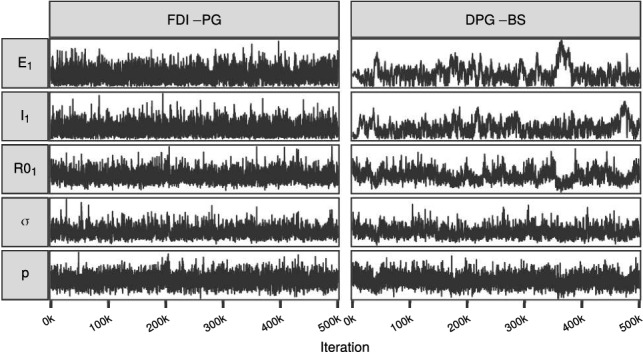
Traceplots for the initial states and model parameters for the SEIR model with the FDI-PG and the DPG-BS. The 5000 samples shown per method and parameter correspond to every 100th sample of the total 500000 samples simulated

We ran both the FDI-PG and the DPG-BS with $N = 64$ a total of $n=500,000$ iterations plus 10, 000 burn-in, and thinning of 10. Figures 10 and 11 show the first 50 autocorrelations and traceplots of $E_1$, $I_1$, $({{\mathscr {R}}_{0}})_1$, $\sigma$ and *p*, for both methods, respectively. The corresponding ${\mathrm {IACT}}$ and ${\mathrm {n}}_{\mathrm {eff}}$ as well as credible intervals for the means of these variables are shown in Table 1. The FDI-PG outperformed the DPG-BS with each variable. However, as is seen from Online Resource 1 (Fig. 4), the difference is most notable with the initial states, and the relative performance of the DPG-BS approaches that of the FDI-PG with increasing state index. The slow improvement in the mixing of the state variable *R* occurs because of the cumulative nature of the variable in the model, and the slow mixing of early values of *I*. We note that even though the mixing with the DPG-BS was worse, the inference with 500, 000 iterations leads in practice to similar findings. However, the FDI-PG could provide reliable inference with much less iterations than the DPG-BS. The marginal density estimates of the initial states and parameters are shown in Online Resource 1 (Fig. 5). The slight discrepancies in the density estimates of $E_1$ and $I_1$ between the methods are likely because of the poor mixing of these variables with the DPG-BS.

**Table 1:** The integrated autocorrelation time, effective sample size and credible intervals of the mean for the initial states and parameters in the SEIR model

Variable	${\mathrm {IACT}}$		${\mathrm {n}}_{\mathrm {eff}}$		95% mean CI	
	FDI-PG	DPG-BS	FDI-PG	DPG-BS	FDI-PG	DPG-BS
$E_1$	30.087	882.583	1661.838	56.652	(353.888, 374.054)	(301.379, 423.106)
$I_1$	14.296	626.963	3497.603	79.75	(165.697, 172.388)	(155.374, 203.458)
$({{\mathscr {R}}_{0}})_1$	32.168	436.755	1554.331	114.481	(3.41, 3.513)	(3.266, 3.636)
$\sigma$	41.261	114.919	1211.796	435.088	(0.15, 0.154)	(0.147, 0.154)
*p*	5.18	38.178	9652.794	1309.647	(0.134, 0.135)	(0.133, 0.135)

**Fig. 12:**
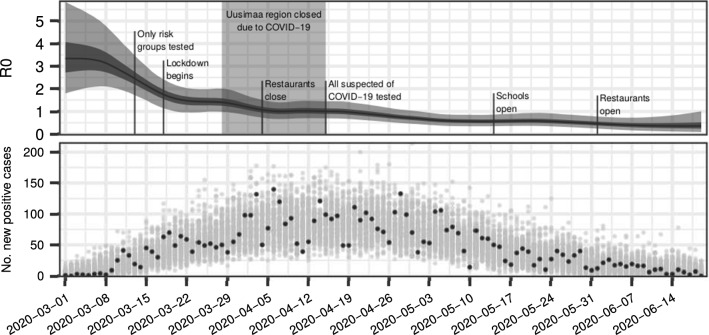
The distribution of the basic reproduction number ${{\mathscr {R}}_{0}}$ (top) and a posterior predictive simulation (bottom) based on the posterior distribution computed with the FDI-PG. The plot for ${{\mathscr {R}}_{0}}$ shows the median in black and probability intervals (75% and 95%) in shades of grey. The black points in the bottom plot represent the data used. The grey points represent observations simulated conditional on the posterior distribution of the model parameters and states

We conclude with a few words about our findings regarding the changing transmission rate, which may be of some independent interest. Figure 12 displays the data and a posterior predictive simulation, and the estimated distribution of ${{\mathscr {R}}_{0}}$ computed by the FDI-PG with respect to time, with annotations about events that may have had an effect on the spread of the epidemic, and/or the data. The initial ${{\mathscr {R}}_{0}}$ is likely somewhat overestimated, because of the influx of infections from abroad, which were not explicitly modelled. There is an overall decreasing trend since the beginning of ‘lockdown’, that is, when the government introduced the first mitigation actions, including school closures. Changes in the testing criteria likely cause some bias soon after the change, but no single action or event stands out.

Interestingly, if we look at our analysis, but restrict our focus up to the end of April, we might be tempted to quantify how much certain mitigation actions contribute to the suppression of the transmission rate in order to build projections using scenario models (cf. Anderson et al. 2020). However, when the mitigation measures have been gradually lifted by opening the schools and restaurants, the openings do not appear to have had notable consequences, at least until now. It is possible that at this point, the number of infections was already so low, that it has been possible to test all suspected cases and trace contacts so efficiently, and that nearly all transmission chains have been contained. Also, the public may have changed their behaviour, and are now following the hygiene and social distancing recommendations voluntarily. Such a behaviour is, however, subject to change over time.

## Discussion

We presented a simple general auxiliary variable method for the CPF for HMMs with diffuse initial distributions and focused on two concrete instances of it: the FDI-CPF for a uniform initial density $M_1$ and the DGI-CPF for a Gaussian $M_1$. We introduced two mechanisms to adapt the FDI-CPF automatically: the adaptive Metropolis (AM) of Haario et al. (2001) and a method similar to a Rao–Blackwellised adaptive scaling within adaptive Metropolis (ASWAM) (cf. Andrieu and Thoms 2008), and provided a proof of their consistency. We also suggested an adaptation for the DGI-CPF, based on an acceptance rate optimisation. The FDI-CPF or the DGI-CPF, including their adaptive variants, may be used directly within a particle Gibbs as a replacement for the standard CPF.

Our experiments with a noisy random walk model and a stochastic volatility model demonstrated that the DGI-CPF and the FDI-CPF can provide orders of magnitude speed-ups relative to a direct application of the CPF and to diffuse initialisation using particle Gibbs, respectively. Improvement was substantial also in our motivating practical example, where we applied the adaptive FDI-CPF (within particle Gibbs) in the analysis of the COVID-19 epidemic in Finland, using a stochastic ‘SEIR’ compartment model with changing transmission rate. Latent compartment models are, more generally, a good example where our approach can be useful: there is substantial uncertainty in the initial states, and it is difficult to design directly a modified model that leads to efficient inference.

Our adaptation schemes are based on the estimated covariance matrix and a scaling factor which can be adapted using acceptance rate optimisation. For the latter, we found empirically that with a moderate number of particles, good performance was often reached with a target acceptance rate ranging in 0.7–0.9. We emphasise that even though we found this ‘0.8 rule’ to work well in practice, it is only a heuristic, and the optimal target acceptance rate may depend on the model of interest. Related to this, we investigated how the optimal target acceptance rate varied as a function of the number of particles and state dimension in a multivariate normal model, but did not find a clear pattern. Theoretical verification of the acceptance rate heuristic, and/or development of more refined adaptation rules, is left for future research. We note that while the AM adaptation performed well in our limited experiments, the ASWAM may be more appropriate when used within particle Gibbs (cf. Vihola 2020). The scaling of the AM remains similarly challenging, due to the lack of theory for tuning.

### Supplementary Information

Below is the link to the electronic supplementary material.

Supplementary material 1 (pdf 137 KB)

## Data Availability

All data analysed in this work are either freely available or available at https://nextcloud.jyu.fi/index.php/s/zjeiwDoxaegGcRe.

## Proof of Theorem 1

For a finite signed measure $\xi$, the total variation of $\xi$ is defined as $\Vert \xi \Vert _{\mathrm {tv}} = \sup _{\Vert f\Vert _\infty \le 1} \xi (f)$, where $\Vert f\Vert _\infty = \sup _x |f(x)|$, and the supremum is over measurable real-valued functions *f*, and $\xi (f) = \int f {\mathrm {d}}\xi$. For Markov transitions *P* and $P'$, define $d(P,P') = \sup _x \Vert P(x,\,\cdot \,) - P'(x,\,\cdot \,) \Vert _{\mathrm {tv}}$.

In what follows, we adopt the following definitions:

### Definition 1

Consider Lines 3 and 4 of Algorithm 1 with $\tilde{X}_1^{(1:N)}=\tilde{x}_1^{(1:N)}$ and ${\dot{x}}_{2:T}$, and define:

(i) $P_{\mathrm {CPF}}(\tilde{x}_1^{(1:N)},{\dot{x}}_{2:T}; \,\cdot \,)$ as the law of $\tilde{X}_{1:T}^{(B_{1:T})}$, and
(ii) (In case PickPath-BS is used:) $\tilde{P}_{\mathrm {CPF}}(\tilde{x}_1^{(1:N)},{\dot{x}}_{2:T}; \,\cdot \,)$ as the law of $\big (\tilde{X}_{1:T}^{(B_{1:T})}, (B_{1},V^{(1:N)}, \tilde{X}_{1}^{(1:N)})\big )$.

Consider then Algorithm 1 with parameterised $Q=Q_\zeta$, and define, analogously:

(iii) $P_\zeta$ is the Markov transition from ${\dot{x}}_{1:T}$ to $\tilde{X}_{1:T}^{(B_{1:T})}$.
(iv) $\tilde{P}_\zeta$ is the Markov transition from from $({\dot{x}}_{1:T},\,\cdot \,)$ to $\big (\tilde{X}_{1:T}^{(B_{1:T})}, (B_{1},V^{(1:N)}, \tilde{X}_{1}^{(1:N)})\big )$.

### Lemma 1

We have $d(P_\zeta , P_{\zeta '}) \le N d(Q_\zeta , Q_{\zeta '})$ and $d(\tilde{P}_\zeta , \tilde{P}_{\zeta '}) \le N d(Q_\zeta , Q_{\zeta '})$.

### Proof

Let $(\hat{P}_\mathrm {CPF}, \hat{P}_\zeta ) \in \{(P_\mathrm {CPF}, P_\zeta ),(\tilde{P}_\mathrm {CPF},\tilde{P}_\zeta )\}$ and take measurable real-valued function *f* on the state space of $\hat{P}_\zeta$ with $\Vert f\Vert _\infty =1$.

We may write

10 $$\begin{aligned}&\hat{P}_\zeta ({\dot{x}}_{1:T}, f) \nonumber \\&= \int Q_\zeta ({\dot{x}}_1,{\mathrm {d}}x_0) \bigg [\int \delta _{{\dot{x}}_1}({\mathrm {d}}\tilde{x}_1^{(1)})\nonumber \\&\qquad \prod _{k=2}^N Q_\zeta (x_0,{\mathrm {d}}\tilde{x}_1^{(k)}) \hat{P}_{\mathrm {CPF}}(\tilde{x}_{1}^{(1:N)}, {\dot{x}}_{2:T}, f)\bigg ], \end{aligned}$$

and therefore, upper bound

$$\begin{aligned}&| \hat{P}_\zeta ({\dot{x}}_{1:T}, f) - \hat{P}_{\zeta '}({\dot{x}}_{1:T}, f) | \\&\quad \le |Q_\zeta ({\dot{x}}_1,g_0^{({\dot{x}}_{1:T})}) - Q_{\zeta '}({\dot{x}}_1,g_0^{({\dot{x}}_{1:T})})| \\&\qquad + \sum _{i=2}^N \int Q_{\zeta '}({\dot{x}}_1,{\mathrm {d}}x_0) | Q_\zeta (x_0, g_i^{({\dot{x}}_{1:T},x_0)} ) \nonumber \\&\qquad - Q_{\zeta '}(x_0, g_i^{({\dot{x}}_{1:T},x_0)} ) | \end{aligned}$$

with functions defined below, which satisfy $\Vert g_0^{({\dot{x}}_{1:T})}\Vert _\infty \le 1$ and $\Vert g_i^{({\dot{x}}_{1:T},x_0)}\Vert _\infty \le 1$:

$$\begin{aligned} g_{0}^{({\dot{x}}_{1:T})}(x_0)= & {} \int \delta _{{\dot{x}}_1}({\mathrm {d}}\tilde{x}_1^{(1)})\prod _{k=2}^N Q_\zeta (x_0,{\mathrm {d}}\tilde{x}_1^{(k)})\\&\quad P_{\mathrm {CPF}}(\tilde{x}_{1}^{(1:N)}, {\dot{x}}_{2:T}, f), \\ g_i^{({\dot{x}}_{1:T},x_0)}(\tilde{x}_1^{(i)})= & {} \delta _{{\dot{x}}_1}({\mathrm {d}}\tilde{x}_1^{(1)}) \prod _{k=2}^{i-1} Q_{\zeta '}(x_0,{\mathrm {d}}\tilde{x}_1^{(k)}) \\&\prod _{k=i+1}^N Q_\zeta (x_0,{\mathrm {d}}\tilde{x}_1^{(k)}) P_{\mathrm {CPF}}(\tilde{x}_{1}^{(1:N)}, {\dot{x}}_{2:T}, f). \end{aligned}$$

$$\square $$

The following result is direct:

### Lemma 2

Let $Q_\varSigma$ stand for the random walk Metropolis type kernel with increment proposal distribution $q_\varSigma$, and with target function $M_1\ge 0$, that is, a transition probability of the form:

$$\begin{aligned}&Q_\varSigma (x,A) = \int _A q_\varSigma ({\mathrm {d}}z) \min \bigg \{1, \frac{M_1(x+z)}{M_1(x)}\bigg \} \\&\quad + 1(x\in A)\bigg (1 - \int q_\varSigma ({\mathrm {d}}z) \min \bigg \{1, \frac{M_1(x+z)}{M_1(x)}\bigg \}\bigg ). \end{aligned}$$

Then, $$\Vert Q_\varSigma (x,\,\cdot \,) - Q_{\varSigma '}(x,\,\cdot \,)\Vert _\mathrm {tv} \le 2 \Vert q_\varSigma - q_{\varSigma '}\Vert _\mathrm {tv}$$.

The following result is from (Vihola 2011, proof of Proposition 26):

### Lemma 3

Let $q_\varSigma (x,{\mathrm {d}}y)$ stand for the centred Gaussian distribution with covariance $\varSigma$, or the centred multivariate *t*-distribution with shape $\varSigma$ and some constant degrees of freedom $\nu >0$. Then, for any $0<b_\ell<b_u<\infty$ there exists a constant $c=c(b_\ell ,b_u)<\infty$ such that for all $\varSigma ,\varSigma '$ with all eigenvalues within $[b_\ell ,b_u]$,

$$\begin{aligned} \Vert q_\varSigma - q_{\varSigma '}\Vert _\mathrm {tv} \le c \Vert \varSigma - \varSigma ' \Vert , \end{aligned}$$

where the latter stands for the Frobenius norm in $\mathbb {R}^d$.

### Assumption 2

(Mixing) The potentials are bounded:

(i) $$\Vert G_k\Vert _\infty <\infty $$ for all $$k=1,\ldots ,T$$.

Furthermore, there exists $\epsilon >0$ and probability measures $\nu _{\zeta }$ such that for all $\zeta \in {\mathsf {Z}}$:

(ii) $Q_\zeta (x_0,A) \ge \epsilon \nu _{\zeta }(A)$ for all $x_0\in \mathsf {X}$ and measurable $A\subset \mathsf {X}$.
(iii) $$\int \nu _{\zeta }({\mathrm {d}}x_0) Q_\zeta (x_0, {\mathrm {d}}x_1) G_1(x_1) \prod _{k=2}^T M_k(x_{k-1}, {\mathrm {d}}x_k) G_k(x_{k-1},x_k) {\mathrm {d}}x_{1:T}$$ $$\ge \epsilon $$.

### Lemma 4

Suppose that Assumption 2 holds, then the kernels $P_{\zeta }$ and $\tilde{P}_\zeta$ satisfy simultaneous minorisation conditions, that is, there exists $\delta >0$ and probability measures $\nu _\zeta ,\tilde{\nu }_\zeta$, such that

$$\begin{aligned} P_{\zeta }^k(x_{1:T},\,\cdot \,) \ge \delta \nu _\zeta (\,\cdot \,) \qquad \text {and}\qquad \tilde{P}_{\zeta }^k(\tilde{x}_{1:T},\,\cdot \,) \ge \delta \tilde{\nu }_\zeta (\,\cdot \,), \end{aligned}$$

for all $x_{1:T}\in {\mathsf {X}}$, $\tilde{x}_1^{(1:N)}\in {\mathsf {X}}^N$, and $\zeta \in {\mathsf {Z}}$.

### Proof

For $\hat{P}_\zeta \in \{P_\zeta , \tilde{P}_\zeta \}$, we may write as in the proof of Lemma1

$$\begin{aligned} \hat{P}_{\zeta }(x_{1:T},\,\cdot \,) = \int Q_\zeta (x_1, {\mathrm {d}}x_0) \hat{P}^*_{\text {CPF},\zeta ,x_0}(x_{1:T},\,\cdot \,), \end{aligned}$$

where the latter term refers to the term in brackets in (10) — the transition probability of a conditional particle filter, with reference $x_{1:T}$, and the Feynman–Kac model $\check{M}_1^{(\zeta ,x_0)}({\mathrm {d}}x_1) = Q_\zeta (x_0, {\mathrm {d}}x_1)$, $M_{2:T}$ and $G_{1:T}$, whose normalised probability we call $\pi ^*_{\zeta ,x_0}$. Assumption 2, 2 and 2 guarantee that $P^*_{\text {CPF},\zeta ,x_0}(x_{1:T},{\mathrm {d}}x'_{1:T}) \ge \varepsilon \pi ^*_{\zeta ,x_0}({\mathrm {d}}x'_{1:T})$, where $\hat{\epsilon }>0$ is independent of $x_0$ and $\zeta$ (Andrieu et al. 2018, Corollary 12). Note that the same conclusion holds also with backward sampling, because it is only a further Gibbs step to the standard CPF. Likewise, in case of $\tilde{P}_\zeta$, the result holds because we may regard $\tilde{P}_\zeta$ as an augmented version of $P_\zeta$ (e.g. Franks and Vihola 2020). We conclude that

$$\begin{aligned} \hat{P}_{\zeta }(x_{1:T},\,\cdot \,) \ge \epsilon \hat{\epsilon } \int \nu _\zeta ({\mathrm {d}}x_0) \pi ^*_{\zeta ,x_0}(\,\cdot \,), \end{aligned}$$

where the integral defines a probability measure independent of $x_{1:T}$. $\square$

We may write the *k*:th step of Algorithm 5 as:

(i) $$(X_k,\xi _k) \sim \tilde{P}_{\zeta _{k-1}}(X_{k-1},\,\cdot \,)$$,
(ii) $$\zeta _k^* = \zeta _{k-1} + \eta _k H(\zeta _{k-1}, X_k, \xi _k )$$,

where *H* correspond to Algorithm 6 or , respectively. The stability may be enforced by introducing the following optional step:

(iii) $$\zeta _k = \zeta _k^* 1(\zeta _k\in {\mathsf {Z}}) + \zeta _{k-1} 1(\zeta _k^*\notin {\mathsf {Z}})$$,

which ensures that $\zeta \in {\mathsf {Z}}$, the feasible set for adaptation.

### Proof

(Proof of Theorem 1) The result follows by (Saksman and Vihola 2010, Theorem 2), as (A1) is direct, Lemma 4 implies (A2) with $V\equiv 1$, $\lambda _n=0$, $b_n=1$, $\delta _n=\delta$ and $\epsilon =0$, Lemmas 2 and 3 imply (A3), and (A4) holds trivially, as $\Vert H (\,\cdot \,)\Vert _\infty < \infty$, thanks to the compactness of *D*. $\square$

## Details of the DPG-BS algorithm

The diffuse particle Gibbs algorithm targets (2) by alternating the sampling of $x_{2:T}$ given $x_1$, and $x_1$ given $x_{2:T}$. Hence, the algorithm is simply particle Gibbs where the initial state is treated as a parameter. Define

$$\begin{aligned} p^{\mathrm {DPG}}(x_1 \mid x_{2:T}) \propto M_1(x_1)G_1(x_1)M_2(x_2 \mid x_1)G_2(x_1, x_2). \end{aligned}$$

With this definition, the DPG-BS algorithm can be written as in Algorithm 10. Lines 3–5 constitute a CPF-BS update for $x_{2:T}$, and Line 6 updates $x_1$. A version of the RAM algorithm (Vihola 2012) (Algorithm 11) is used for adapting the normal proposal used in sampling $x_1$ from $p^{\mathrm {DPG}}$.
