# Supplementary material for: Conditional particle filters with diffuse initial distributions
Source: Stat Comput. 2021 Mar 3;31(3):24. doi: 10.1007/s11222-020-09975-1 (PMC7926083; doi:10.1007/s11222-020-09975-1)
Supplement: Supplementary file 1 — Supplementary material 1 (pdf 137 KB) [file 11222_2020_9975_MOESM1_ESM.pdf]

Supplementary figures for 'Conditional particle filters with diffuse initial distributions'

Statistics and Computing

Santeri Karppinen (corresponding author, email: skarppinen@iki.fi) and Matti Vihola

University of Jyväskylä, Department of Mathematics and Statistics, Jyväskylä, FI-40014, Finland

November 20, 2020

Supplementary Figures

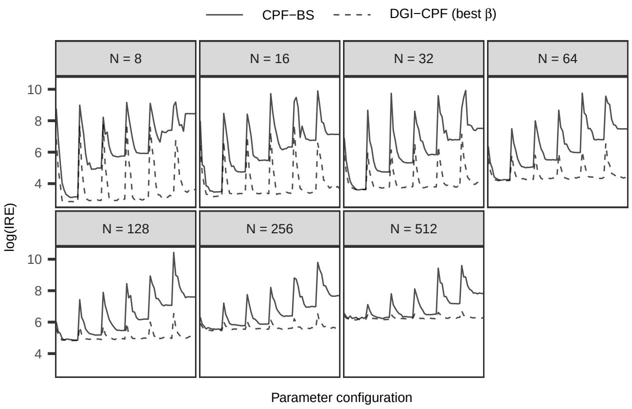

Figure 1: The log (IRE) resulting from the application of the CPF-BS and the best case DGI-CPF to the SV model. The horizontal axis depicts different configurations of σ_1 and σ_x, and in each panel N varies.

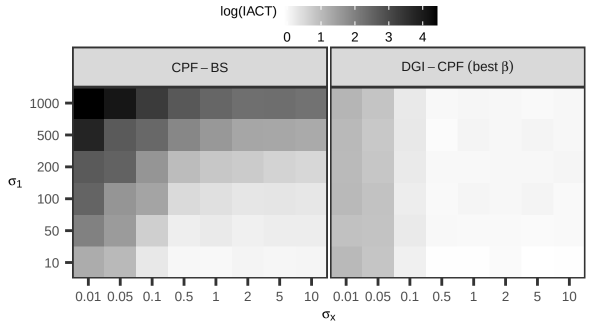

Figure 2: The log (IACT) of the CPF-BS (left) and the best case DGI-CPF (right) with respect to σ_1 and σ_x in the case of the RW model and $N = 256$.

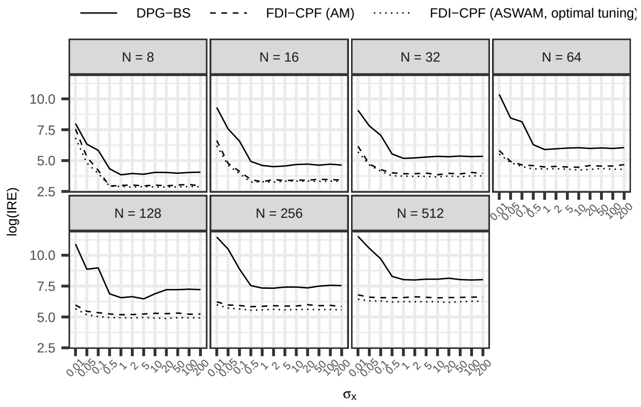

Figure 3: The log (IRE) for the DPG-BS, the FDI-CPF with the AM adaptation and the best case FDI-CPF with the ASWAM adaptation to the datasets generated with varying σ_x from the SV model.

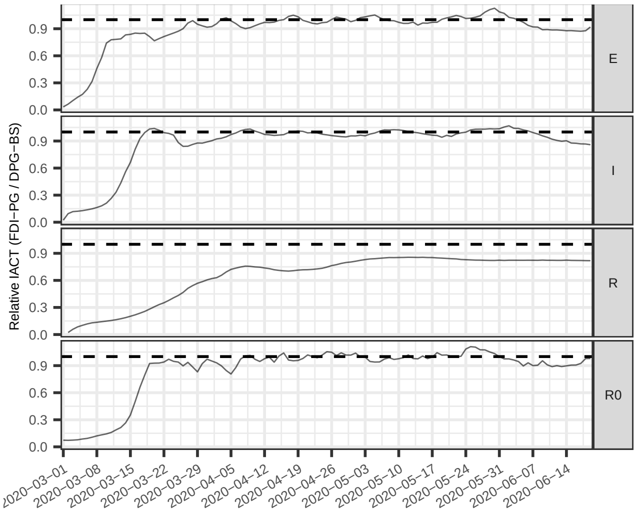

Figure 4: The integrated autocorrelation time with the FDI-PG relative to that of the DPG-BS for the state variables at each time point in the SEIR model. The dashed line shows the line of equal sampling efficiency. The first value for the state variable R is missing, since $R_1 = 0$ is assumed in the model.

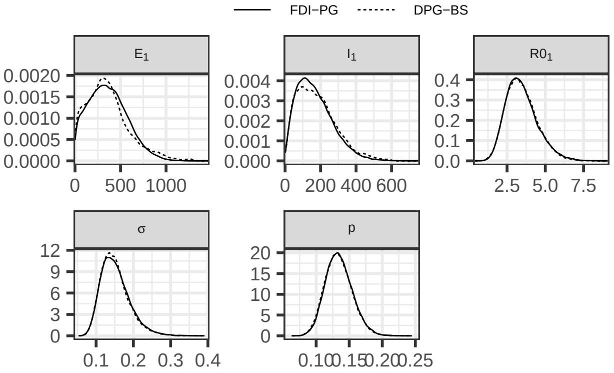

Figure 5: Marginal density estimates of the initial states and parameters for the SEIR model computed by the FDI-PG and DPG-BS.
